# Effect of Microcapsule Content on Diels-Alder Room Temperature Self-Healing Thermosets

**DOI:** 10.3390/polym12123064

**Published:** 2020-12-21

**Authors:** Sadella C. Santos, John J. La Scala, Giuseppe R. Palmese

**Affiliations:** 1 Department of Chemical & Biological Engineering, Drexel University, 3141 Chestnut Street, Philadelphia, PA 19104, USA; sadellasantos1@gmail.com; 2 Army Research Laboratory, 4600 Deer Creek Loop, Aberdeen Proving Grounds, MD 21005-5096, USA; john.j.lascala.civ@mail.mil

**Keywords:** microencapsulation, self-healing polymers, Diels-Alder, diffusion, plasticization

## Abstract

A furan functionalized epoxy-amine thermoset with an embedded microcapsule healing system that utilizes reversible Diels-Alder healing chemistry was used to investigate the influence of microcapsule loading on healing efficiency. A urea-formaldehyde encapsulation technique was used to create capsules with an average diameter of 150 µm that were filled with a reactive solution of bismaleimide in phenyl acetate. It was found that optimum healing of the thermoset occurred at 10 wt% microcapsule content for the compositions investigated. The diffusion of solvent through the crack interface and within fractured samples was investigated using analytical diffusion models. The decrease in healing efficiency at higher microcapsule loading was attributed partially to solvent-induced plasticization at the interface. The diffusion analysis also showed that the 10% optimum microcapsule concentration occurs for systems with the same interfacial solvent concentration. This suggests that additional physical and chemical phenomena are also responsible for the observed optimum. Such phenomena could include a reduction in surface area available for healing and the saturation of interfacial furan moieties by reaction with increasing amounts of maleimide. Both would result from increased microcapsule loading.

## 1. Introduction

Autonomous self-healing materials are materials that can repair damage to themselves or recover a functionality without external intervention [1]. Self-healing thermosets are desirable for recovery of mechanical strength through repair of damage due to wear or internal fractures resulting from fatigue. The modes of self-healing have been classified into three categories: intrinsic, vascular, and capsule based [1,2,3].

**Figure 1:**
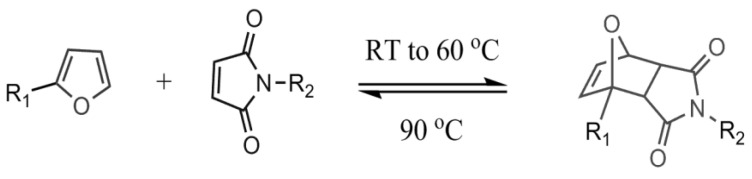
Diels-Alder reaction between furan and maleimide.

The Diels-Alder reaction has been widely used for self-healing chemistry because certain forms of the reaction are thermoreversible, where with the addition of heat causes the formed adduct to revert to the original diene and dienophile. Chen et al. [4] developed one of the initial self-healing thermosets using reversible Diels-Alder chemistry in which cross-links were formed between furans and maleimides as described below in Figure 1.

Peterson et al. developed a healable furan-functionalized epoxy-amine thermoset based on the Diels-Alder reaction [5,6,7]. A monomer containing a furan group was added to an epoxy-amine mixture, resulting in a furan-functionalized thermosetting network. Reactive solutions of bismaleimide and solvent were applied directly to the fractured surfaces and were allowed to heal at room temperature and under minimal pressure. It was found that in these systems, that the solvent induces swelling, thus bringing fracture surfaces together and delivering maleimide functional healing agents that form covalent bonds across the crack [5].

Pioneering work on capsule-based self-healing thermosetting networks was done by White et al. [8,9] Microcapsules filled with reactive solutions of dicyclopentadiene (DCPD) and Grubbs’ catalyst were dispersed throughout a thermoset. Upon fracture, the capsules break and release DCPD into the crack interface, where it can react with the catalyst and subsequently undergo ring opening metathesis polymerization (ROMP) to bind the crack faces closed.

Pratama et al. developed a capsule-based healing system [10] for the aforementioned furan-functionalized network that utilizes reversible Diels-Alder reversible chemistry shown in Figure 1. Capsules filled with reactive solutions of bismaleimide and solvent were dispersed throughout the polymer network [7,10,11]. In response to cracks within the system, these capsules break to release reactive solution to the crack interface, allowing covalent bonds to form between the maleimide and furans and bind the crack faces together. Initial studies showed that with the addition of 10 wt% maleimide solution-filled capsules, the resulting thermoset recovered approximately 71% of the initial load [10].

In this work we investigate the influence of microcapsule concentration on healing efficiency of the Diels-Alder room temperature capsule-based self-healing systems. Microcapsules filled with solutions of bismaleimide in phenyl acetate with an average diameter of 150 μm were prepared and a furan-functionalized polymer epoxy network based on diglycidyl ether of bisphenol A and furfuryl glycidyl ether was used to make thermoset bars containing varying amounts of the microcapsules. Room temperature healing studies were conducted using compact tension specimens with arrested cracks and the effects of Diels-Alder reaction kinetics and solvent diffusion on network properties and healing efficiency were studied.

## 2. Experimental Methods and Materials

### 2.1. Materials

Diglycidyl ether of bisphenol A (DGEBA with EEW = 185–192, EPON 828, Miller-Stephenson, Danbury, CT, USA), furfurylglycidyl ether (FGE, Sigma-Aldrich, 96%, St. Louis, MO, USA), and 4,4′-methylene biscyclohexanamine (PACM with AEW = 52.5, Evonik, Allentown, PA, USA) were used to prepare furan-functionalized epoxy amine thermosets. Fumed silica (Cab-o-sil, Cabot, Boston, MA, USA) was used as a rheology modifier. The healing solution was obtained by dissolving MMI-2,1,6′-bismaleimide-(2,2,4-trimethyl)hexane (BMI-TMH Daiwakasei Industries, Parisppany, NJ, USA), in phenyl acetate (99% Sigma-Aldrich). The chemical structures of the monomers, solvents and maleimide healing agents are shown in Table 1. Urea, ammonium chloride, resorcinol, 36% formaldehyde solution, and 2 M aqueous sodium hydroxide used for microcapsule synthesis were obtained from Sigma-Aldrich. The surfactant used for microcapsule synthesys was ethyl maleic anhydride (EMA) polymer (Zemac E-400, Vertellus Holdings LLC, Indianapolis, IN, USA). All materials were used without additional purification.

### 2.2. Microcapsule Preparation

Microcapsules containing a bismaleimide compound in phenyl acetate (PA) solution were prepared by in situ polymerization of urea and formaldehyde, using the process outlined by Pratama et al. [10] PA was chosen as the core solvent because of a preferential solubility of maleimide and its ability to sufficiently swell the polymer matrix. Based on the results of previous work [7,10,11], MMI-2 bismaleimide was used for encapsulation. Concentration of surfactant (EMA, Zemac E-400) was optimized to prevent agglomeration and to ensure capsule stability [10]. The reaction was allowed to progress for 4 h under constant heating and mechanical agitation. At completion of the reaction, mechanical agitation was continued for an additional 6 h at room temperature. Multiple washes using water and acetone were performed, and centrifugation was used to separate the capsules from the aqueous phase. After a drying period of 24 h, the capsules were characterized and processed before incorporation into the furan functional epoxy.

### 2.3. Microcapsule Characterization

Scanning electron microscopy (SEM, Zeiss Supra 50VP, Carl Zeiss Microscopy LLC , White Plains, NY, USA) coupled with ImageJ (National Institute of Health) was used to characterize the capsule shell thickness, agglomeration, and average microcapsule size. The SEM samples were sputter coated with platinum, and measurements were made at 5.0 kV accelerating voltage.

Thermogravimetric analysis (TGA, TA Instruments TAQ50, TA Instruments, New Castle, DE, USA) was used to determine MMI-2/PA content within microcapsules [10]. The amount of encapsulated solution is taken as the mass loss during the TGA run. Capsule specimens were heated at a rate of 5 °C min^−1^ to 100 °C and held for 30 min to stimulate water loss. This was followed by heating to 180 °C for 30 min and 200 °C for 60 min, which bracketed the boiling point of PA (b.p. 196 °C).

### 2.4. Preparation of Capsule-Based Self-Healing Thermoset

For the study of healing efficiency vs. microcapsule loading, a furan-functionalized epoxy-amine thermoset was prepared by mixing a 6:4 by weight ratio of DGEBA and FGE with a stoichiometric amount of PACM curing agent. To avoid settling and to keep microcapsules well dispersed after mixing, 1 wt% fumed silica was added. The addition of the fumed silica is known to have a minimal effect on the mechanical properties of the polymer. Using a THINKY planetary mixer (THINKY U.S.A., Laguna Hills, CA, USA), resins were mixed at 2000 rpm for 4 min and degassed at 800 rpm for 1 min. Microcapsules 200 μm in diameter and less were obtained using a sieve before being hand-mixed into the pre-mixed resin at the appropriate weight percentage. Hand mixing was employed to prevent fracture of the microcapsules. The microcapsule-filled resin was then poured into the appropriate molds and cured for 2 h at 60 °C and for 2 h at 90 °C.

### 2.5. Compact Tension Testing

Values for healing efficiency were calculated using modified compact tension specimens in accordance with ASTM D 5045–99. This commonly used testing procedure for healing efficiency has been previously described for this system by Peterson et al. [5,10] The addition of a crack-arresting hole to the specimens prevents crack propagation through the entire length of the sample and also aids in realignment of crack surfaces during healing. A pre-crack was introduced by running a razor blade across the base of the notch [10]. An Instron 8872 (Instron Corporation, Norwood, MA, USA) was used to apply uniaxial tensile loading, at a loading rate of 0.1 mm min^−1^ to the samples until fracture. Specimens were healed at ambient conditions (~25 °C) under minimal pressure (~50 kPa) for various amounts of time. After healing, samples were again loaded until fracture. The healing efficiency is defined as the ratio of the max load for the initial and healed samples. Healing efficiency for each sample was calculated using Equation (1), where *η* is healing efficiency, *P*_0_ is the maximum load for the initial fracture of the specimen, and P_h_ is the maximum load for the healed specimen.

(1) $$\eta =\frac{P_{h}}{P_{0}}\times 100\%$$

All healing efficiency results are presented as averages of at least five tested specimens with error bars representing standard error of the mean of the population.

### 2.6. Characterization of the Diels-Alder Reaction

Fourier-transform near-infrared spectroscopy (FTNIR) (Nicolet iS50 FT-IR spectrometer, Thermo Fisher Scientific, Waltham, MA, USA) was used to study the reaction kinetics between the maleimide compound in the MMI-2 healing agent and the furan groups within the polymer network. The decrease in characteristic maleimide absorbance at 4875 cm^−1^ and at 25 °C was followed, representing the decrease in maleimide concentration as the forward reaction occurs. Stoichiometric solutions of 2.30 M FGE and 0.26 M of maleimide groups from MMI-2 with PA as the solvent were prepared in sealed glass capillary tubes. These values were chosen to represent the values of furan and maleimide groups in the polymer and capsules, respectively.

### 2.7. Dynamic Mechanical Analysis

Dynamic mechanical analysis (DMA, TA Instruments Q800, TA Instruments, New Castle, DE, USA) was used to study the change in mechanical properties of samples. Rectangular sample bars with dimensions of approximately 35 mm × 12.7 mm × 3.1 mm were prepared. Samples were scanned from −80 °C to 100 °C at a ramp rate of 2 °C min^−1^, at a frequency of 1 Hz, and with a deflection of 15 μm using single cantilever geometry.

## 3. Results and Discussion

### 3.1. Microcapsule Characterization

**Figure 2:**
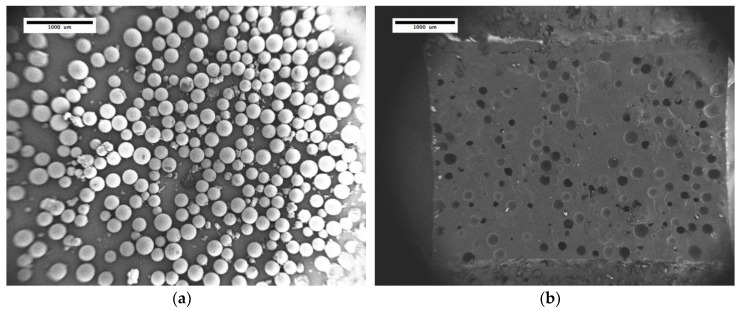
SEM Images of (**a**) dried microcapsules and (**b**) crack interface of resin with 20 wt% microcapsule content. Samples were sputter coated with platinum.

**Figure 3:**
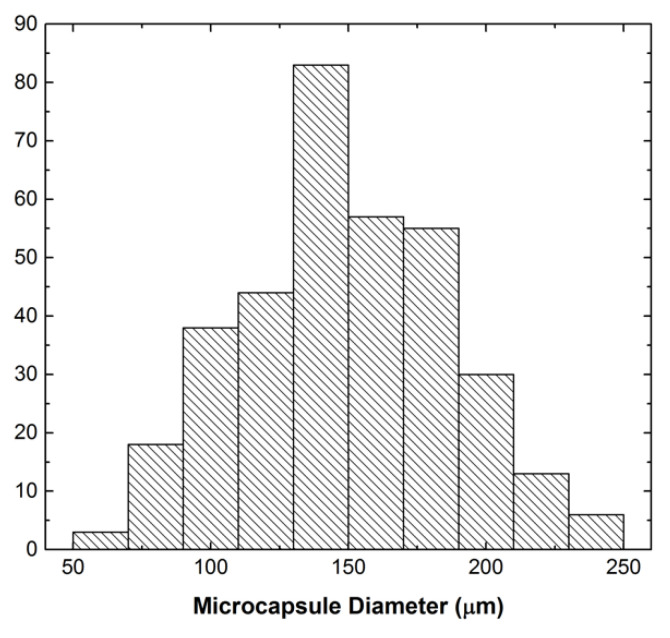
Size distribution of microcapsules filled with PA and MMI-2 healing agent.

Microcapsules filled with MMI-2 reactive solutions were successfully synthesized. Scanning electron microscopy images of microcapsules filled with 0.13 M MMI-2 in PA and the crack interface of compact tension test samples are shown in Figure 2. The capsules produced were spherical in shape and separable from UF debris seen in solution through solvent washing. The resulting microcapsules are not monodisperse; however, it was found that larger capsules could be removed using mesh filters of an appropriate size. Capsules with diameters nominally less that 200 μm were separated and incorporated into the epoxy system. A histogram describing the particle size distribution of these capsules is shown in Figure 3. The average diameter for microcapsules filled with MMI-2 and PA after filtration was 149.9 ± 38.4 µm.

**Figure 4:**
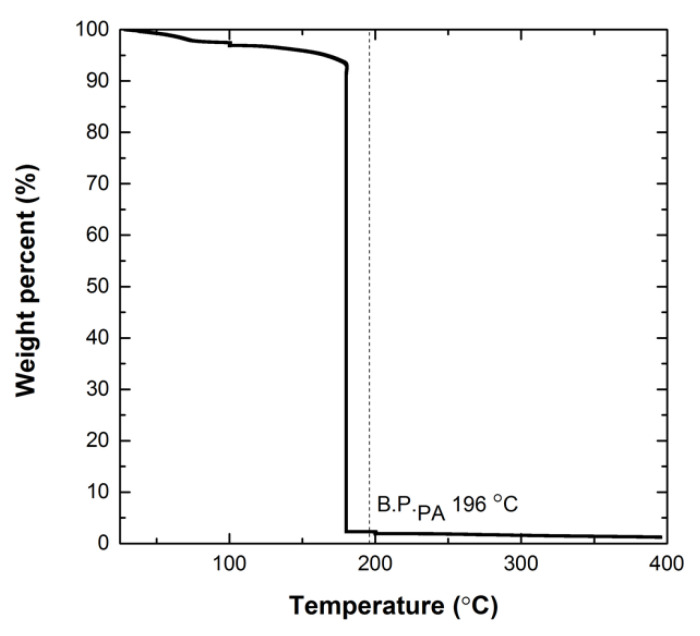
Thermogravimetric analysis (TGA) of microcapsules filled with MMI2/PA. Microcapsules used in TGA have diameters of 200 μm and less. Capsule specimens were heated at a rate of 5 °C min [1] to 100 °C and held for 30 min to stimulate water loss. This was followed by heating to 180 °C for 30 min and 200 °C for 60 min, which bracketed the boiling point of PA (b.p. 196 °C).

A TGA scan of microcapsules filled with MMI2 and PA is shown in Figure 4. Analysis shows an initial slight decrease of the sample weight at 100 °C, which corresponds to residual water and acetone (b.p. 56 °C) on the capsule surface. The decrease in weight percentage between 160–200 °C corresponds to the loss of PA (b.p. 196 °C). The TGA experiments show that the PA content was ~90% by weight.

### 3.2. Characteristic Time for Adduct Formation

**Figure 5:**
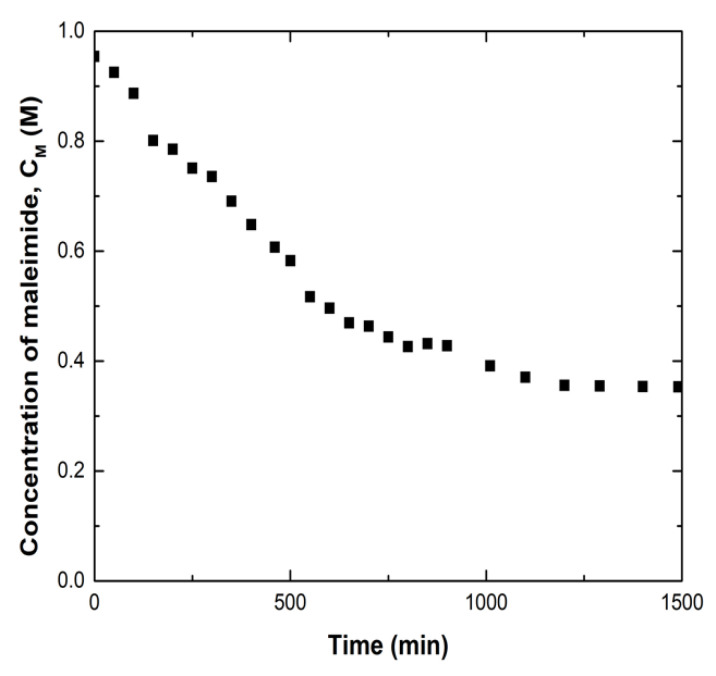
Maleimide concentration during reaction of furan and maleimide in 2.3 M FGE 0.26 M maleimide groups from MMI2 in PA at 25 °C.

A kinetics analysis of the forward Diels-Alder reaction in which the maleimide and furan groups formed an adduct was conducted using 2.3 M FGE and 0.26 M MMI2 in PA solutions at 25 °C. The rate of adduct formation was measured using the absorbance at 4875 cm^−1^, which correlates well to maleimide double bond concentration [5,6,7]. Figure 5 shows maleimide concentration as a function of time for the forward Diels-Alder reaction. In this system, full reaction (reaction approaching equilibrium) was achieved in approximately 1000 min or 16.7 h. Because the reaction reaches equilibrium before this study’s first healing measurement at 48 h, it will be assumed that for all healing time points used in this study, the majority of maleimide-furan bonds at the crack surface have been formed.

### 3.3. Self-Healing via Incorporation of Microcapsules

**Figure 6:**
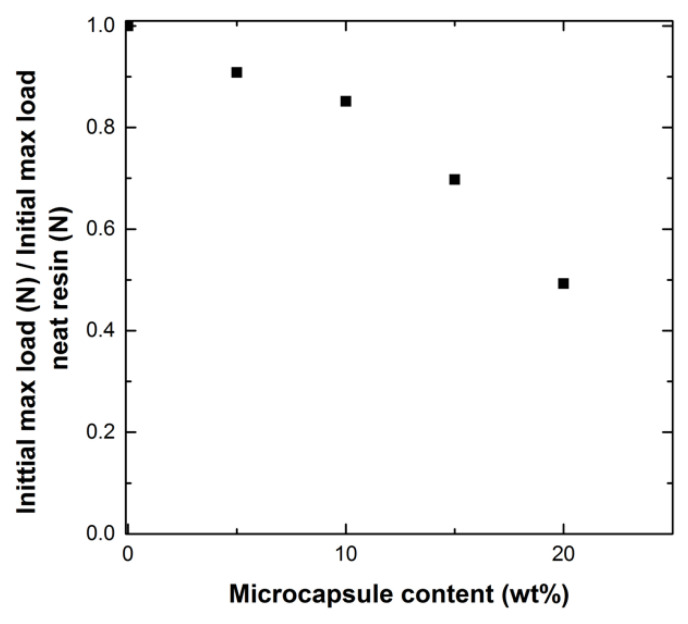
Initial maximum load of fracture (N) of virgin fracture toughness samples (Day 0) normalized to the initial maximum load value (N) of sample of neat resin for specimen with varying microcapsule content (wt%).

Microcapsules were added to the thermoset in varying weight percentages by hand to prevent fracture of the microcapsules. Figure 6 shows the maximum load values (N) of the virgin fracture toughness samples (Day 0). These values were normalized to the maximum load value of the virgin fracture toughness sample with no added microcapsules for ease of comparison. As microcapsules are added, the fracture strength is reduced. Beyond 10 wt% microcapsules, the fracture load drops more rapidly, indicating that microcapsule loadings of 10 wt% or less will be likely favored.

**Figure 7:**
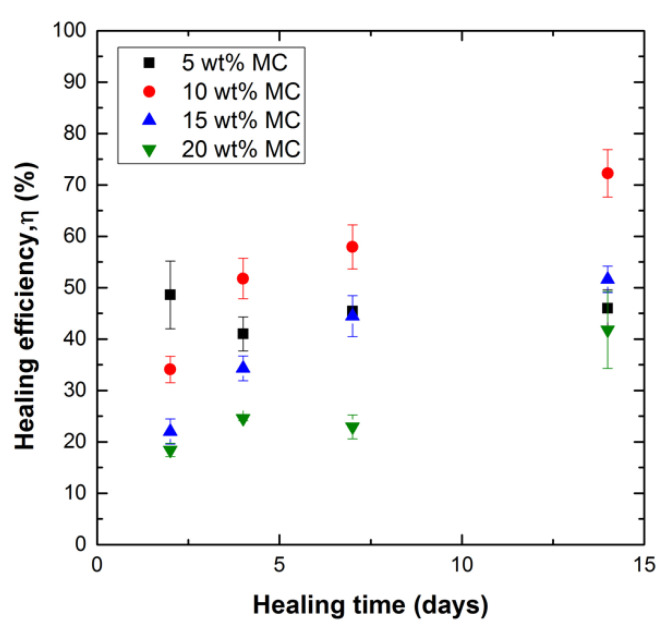
Healing efficiency, η (%) vs. healing time (days) for modified fracture toughness samples with varying microcapsule content. Specimens were healed for 2, 4, 7, and 14 days.

Figure 7 shows the healing efficiency values calculated for samples with varied microcapsule content as a function of healing time. The four sample groups correspond to increasing microcapsule content: 5, 10, 15, 20 wt%. The values for healing efficiency were calculated using Equation (1). The samples were allowed to heal for 2, 4, 7, and 14 days under minimal pressure (~50 kPa). The data show that with the exception of 5 wt% loading, the healing efficiency increases with increased healing time. At 2 days of healing, it was observed that as microcapsule content increases, the healing efficiency decreases. Such behavior is counterintuitive since higher capsule concentration corresponds to more healing agent available for healing. At 4 days healing time and longer, this trend is no longer observed. Figure 7 also shows that for a long healing time (14 days) there is an optimum healing efficiency at 10 wt% microcapsule content, and that increasing microcapsule content to 20 wt% results in a much-reduced healing efficiency.

We hypothesize that although covalent bonds have fully formed at the crack interface (as suggested by the kinetic study), the increased volume of solvent associated with higher capsule concentration leads to more pronounced plasticization of the matrix. This could cause a decrease in T_g_ to a value close to the testing temperature so the polymer is unable to recover its original mechanical properties for the given healing time. The degree to which PA diffusion and plasticization of the polymer are responsible for the observed self-healing behavior were investigated further as described in the following sections.

### 3.4. Solvent Diffusion and Plasticization

**Figure 8:**
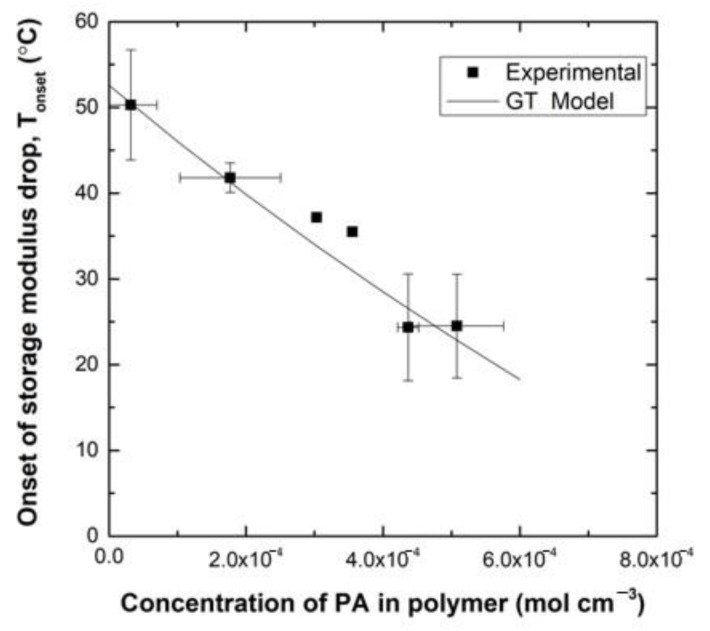
Temperature of the extrapolated onset of the storage modulus change (°C), T_onset_, of DMA specimen with varying concentrations of PA in polymer (M). Data shows that with increasing mass uptake of solvent, T_onset_ decreases.

A plasticization study was conducted to investigate the influence of PA content on the properties of the epoxy network. DMA sample bars of the DGEBA:FGE(6:4)/PACM system were made and individually placed into sealed glass vials of PA, ensuring full immersion in solvent. The initial mass of the specimen was noted before immersion. Bars were left in the vials to allow uptake of varying concentrations of solvent and then subsequently removed and weighed to note the final mass. The specimen was then placed in an empty, sealed glass vial for an extended period to ensure that an equilibrium distribution of solvent throughout the polymer had been reached. Upon reaching equilibrium, DMA was used to measure the extrapolated onset of the storage modulus change, T_onset_, for the varying concentrations of PA in polymer. This value represents the point at which the neat resin system moves from the glassy plateau to the glass transition region—the point at which the material starts to become compliant. The experimental T_onset_ values for varying concentrations of PA in the DMA bars are shown in Figure 8. The experimental data show that with increasing mass uptake of solvent, T_onset_ of the polymer decreases significantly. Changes in T_g_ are related proportionally to changes in T_onset_, thus a relationship between T_g_ and amount of plasticizer can also be used to study the relationship between T_onset_ and the amount of plasticizer.

The Gordon-Taylor equation [12], given by Equation (2), describes the T_g_ of plasticized polymers as a function of weight percent of solvent (w) and T_g_ values, respectively, of the pure solvent, 1, and the polymer, 2. The adjustable parameter k_GT_ can be calculated from the experimental data of Figure 8.

(2) $$T_{g}=\frac{w_{1}T_{g,1}+k_{\mathrm{GT}}w_{2}T_{g,2}}{w_{1}+k_{\mathrm{GT}}w_{2}}$$

The Gordon-Taylor equation was used to fit the aforementioned experimental data and to estimate T_onset_ values for polymer samples with varying weight percentages of PA. The fit is also given in Figure 8 and shows good agreement with the data. Based on this fit it is estimated that a concentration of 4.16 × 10^−4^ mol/cm^3^ (~4.8 wt%) PA to the epoxy network will cause T_onset_ to decrease to 25 °C.

The governing equation for the diffusion of PA through the compact tension sample during healing can be described by the one-dimensional diffusion equation, given by Equation (3). *C_A_* represents the concentration of PA and *D_A_* is the diffusion coefficient of PA in the polymer.

(3) $$\frac{\partial C_{A}}{\partial t}=D_{A}\frac{\partial^{2}C_{A}}{\partial x^{2}}$$

To model the diffusion of PA that occurs in the compact tension specimen, the zone of diffusion was defined as the distance from the crack surface (*x* = 0) to half of the width of the compact tension specimen (*x* = *L*). Upon damage, there is a finite amount of PA that can be delivered to the crack interface from fractured microcapsules. The released volumes of PA (V_c_) for a given fracture surface area (SA) were estimated using SEM micrographs by counting the number of fractured capsules and assuming them to be 90% filled. Furthermore, if a uniform film is formed at the interface, it would have a thickness *l_c_ = V_c_/SA*. Two major assumptions are made to solve this diffusion problem: first, volume changes due to solvent diffusion into the polymer matrix are negligible; second, the diffusion coefficient is constant and does not decrease over time due to the Diels-Alder reaction with the healing agent increasing cross-link density.

To model the diffusion process, two stages were considered, each of which was solved analytically. In the first stage, solvent is released into the crack upon fracture. This volume (V_c_) of solvent increases with increasing capsule content and is assumed to form a uniform film at the crack interface. At this stage, the diffusion of PA can be approximated as diffusion into a semi-infinite slab in which the initial concentration of solvent is *C_A_ =* 0 throughout the sample (0 < *x* < *L*), a no flux boundary condition is found at L (*x = L, ∂C_A_/∂x =* 0), and a constant equilibrium concentration of PA is applied at the crack surface (*x =* 0*, C_A_ = C_eq_*). The analytical solution for this problem is given by Equation (4) [13].

(4) $$C_{A}\left(x,t\right)=C_{eq}\left[1-erf\left(\frac{x}{\sqrt{D_{A}t}}\right)\right]$$

At the point that the solvent film volume is fully absorbed, the boundary condition at the crack interface also becomes no flux (*x =* 0*, ∂C_A_/∂x =* 0). This happens at a time, *t*_fill_, when the amount of PA that has diffused through the system is equal to the initial amount of PA delivered from the fractured microcapsules. This time is found analytically as the time when Equation (5) is satisfied. In this equation, *C_A_*_0_ is the molar concentration of pure PA.

(5) $$C_{A0}l_{c}=\;\int_{0}^{L}C_{A}\left(x,t\right)\;\partial x$$

Based on previous and current diffusion studies, [10] *C*_eq_ = 3.12 × 10^−3^ mol/cm^3^ and D = 7.68 × 10^−9^ cm^2^ s^−1^, and using Equations (4) and (5), values of *t*_fill_ for samples with 5, 10, 15, and 20 wt% capsule content were calculated to be 0.22, 0.50, 0.66, and 1.14 h, respectively.

**Figure 9:**
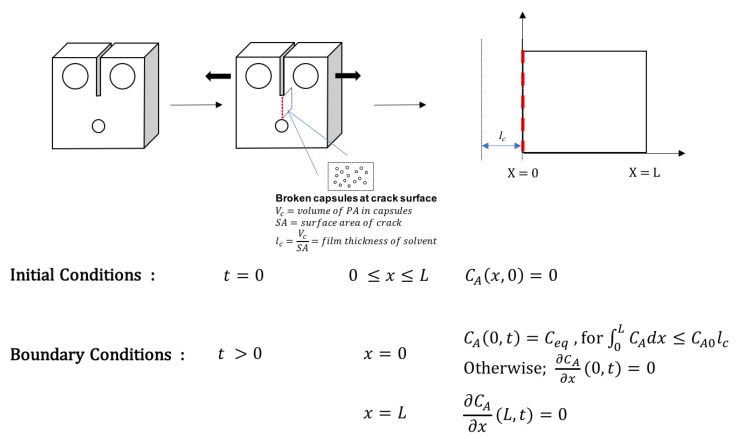
Schematic of model for diffusion of phenyl acetate through compact tension specimen fracture surface.

For the second stage of the diffusion process, when the solvent reservoir has been depleted, no flux boundary conditions are applied at *x* = *L* and *x* = 0. At *x* = 0, this is the case because two symmetrical halves of the fracture surfaces come together. The boundary condition at *x* = *L* is set to no flux for convenience and because the volatility of PA at 25 °C is low. Therefore, the second stage of the diffusion process is approximated by diffusion within a flat sheet, where both surfaces *x* = 0 and *x* = *L* are impermeable, with the initial condition being the concentration profile defined by Equation (4) at *t = t*_fill_. The analytical solution for this problem is given in Equation (6), where *f(x’)* defines the initial concentration profile^13^. A schematic representation of the two-stage model is shown in Figure 9.

(6) $$C_{A}\left(x,t\right)=\frac{1}{L}\int_{0}^{L}f\left(x^{\prime }\right)dx^{\prime }+\;\frac{2}{L}\overset{\infty }{\underset{n=1}{\sum }}\exp\left(-\frac{D_{A}n^{2}\pi^{2}t}{L^{2}}\right)\cos\frac{n\pi x}{L}\int_{0}^{L}f\left(x^{\prime }\right)\cos\frac{n\pi }{L}dx'$$

**Figure 10:**
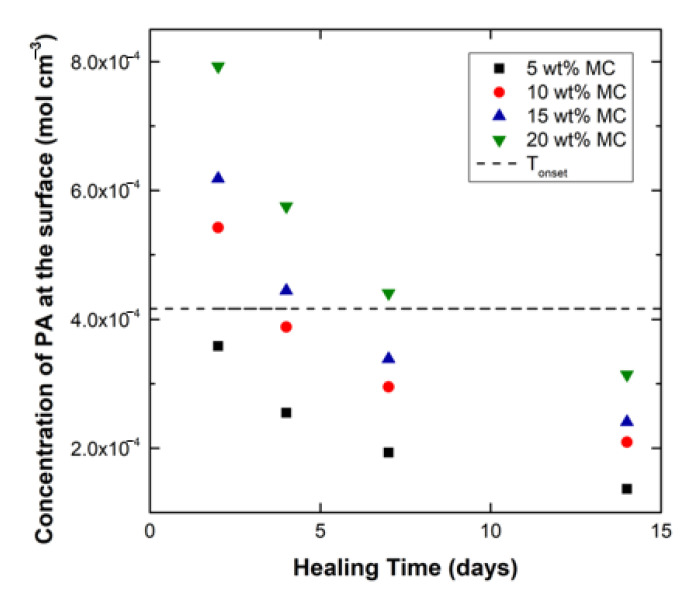
Calculated concentration of PA in the polymer for samples with varying microcapsule content at varied healing times (2, 4, 7, and 14 days). The concentration at which T_onset_ = 25 °C is also plotted.

Equations (4) and (6) were used to solve for PA concentration profiles in the systems having 5, 10, 15, and 20 wt% microcapsules for healing times of 2, 4, 7, and 14 days. The resulting concentration profiles were used to obtain the surface concentration of PA for all the capsule systems at each healing time. The surface solvent concentration values in turn were used in Equation (2) to predict the T_onset_ values at the time of healing and subsequent testing. Using the procedures described above, the calculated concentrations of PA at the crack surface for the four capsule contents at varying healing times are plotted in Figure 10. In addition, the concentration of PA that results in a T_onset_ of 25 °C is shown as a horizontal line on the plot.

The model results suggest that for initial healing times (2–4 days), the samples with higher capsule content (10, 15, and 20 wt%) would be significantly affected by solvent plasticization. After 2 days of healing, the 10, 15, and 20 wt% samples are predicted to have very high concentrations of PA at the crack interface, as they are well above the T_onset_ = 25 °C threshold. This high amount of PA means that the plasticized crack interface of these samples will have a T_onset_ below 25 °C and a T_g_ much lower than that of the cured neat resin. As the healing time progresses to 4 and 7 days and the PA diffuses from the crack throughout the polymer, the 10 and 15 wt% systems move below the threshold line while the 20 wt% samples still contain enough PA to keep them fully plasticized and weak. At 14 days, all systems are below the threshold PA concentration, although the concentration varies proportionally to the capsule content. The healing efficiency of systems with T_onset_ less than 25 °C at the crack interface are predicted to exhibit poor healing efficiency.

**Figure 11:**
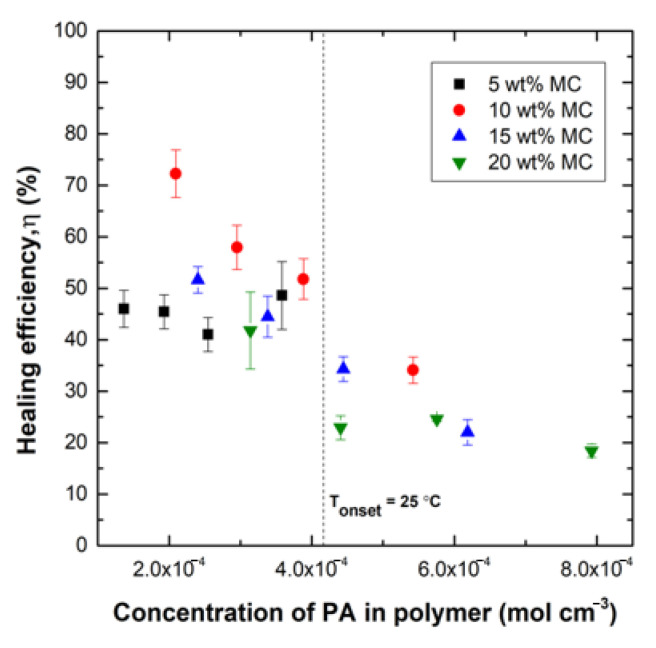
Healing efficiency, η, vs. concentration of PA in the polymer as calculated by the analytical model for specimena with varying microcapsule content at varied healing times (2, 4, 7, and 14 days). The concentration needed for T_onset_ = 25 °C is plotted to differentiate which specimens are affected by plasticization.

The healing efficiency values for all samples were compared to the PA surface concentration values predicted by the analytical model. These values are given in Figure 11 as a plot of healing efficiency versus calculated surface concentration of PA. The concentration needed for T_onset_ = 25 °C is also plotted as a vertical line that can be used to differentiate which specimens would be severely affected by plasticization and which would not. The points to the right of the line are predicted to be highly plasticized and, in fact, show reduced healing efficiencies ranging from 20–35% than the points to the left of the line, which have healing efficiencies ranging from 40–75%. The plot also shows that for 5% loading, the interfacial solvent concentration is lower than the T_onset_ threshold for all healing times.

Figure 11 can also be used to define the sets of points that are not significantly affected by plasticization, allowing for meaningful comparisons to better understand the influence of capsule loading on healing efficiency. Comparisons of the datasets to the left of the T_onset_ concentration show that there is an optimum capsule loading in the range of 10% for these systems that is not a result of plasticization. For the same predicted low concentration of solvent, the healing efficiency for 10% capsule loading is always highest. There are several potential explanations for this result currently under investigation: (1) Greater amounts of maleimide could saturate furan sites at the interface, resulting in less bridging between fracture surfaces; (2) increasing capsule concentration decreases the surface area available for bonding; and (3) more debris is generated with a higher concentration of capsules, which further reduces the available surfaces for bonding. We conclude that the observed optimum capsule concentration is not only a result of the plasticization effect as it remains observable when the solvent dissipates.

## 4. Conclusions

Microcapsules filled with solutions of bismaleimide in phenyl acetate with an average diameter of 150 μm were made. A furan-functionalized polymer network was used to prepare systems containing varying amounts of the MMI2-PA-filled capsules in order to investigate the influence of capsule content on healing efficiency. Results showed that there is an optimum capsule content at which the highest healing efficiency of the epoxy-amine network was achieved. The optimum capsule content was found to be 10 wt% with a healing efficiency of 75% for the range of compositions investigated. Solvent is needed for healing as it promotes fracture surface contact by localized swelling as well as a reaction by reducing diffusion limitations associated with the glassy state; however, the accompanying reduction in T_g_ by plasticization also weakens the interface. Higher capsule loading increases maleimide moieties available for the healing reaction but also the amount of solvent that can remain trapped at the interface, reducing healing efficiency. A diffusion analysis was conducted to estimate the solvent concentration remaining at the interface as a function of healing time and microcapsule loading. Analysis of healing performance as a function of predicted solvent concentration at the fracture interface shows that high capsule loadings lead to greater degrees of plasticization. This is a partial explanation for the observed optimum microcapsule concentration because the analysis also shows that the optimum is observed for systems with the same interfacial solvent concentration. Possible physical and chemical explanations for this include: (1) a reduction in surface area available for healing, and (2) the saturation of interfacial furan moieties by reaction with the higher amounts of maleimide, both resulting from increased microcapsule volume fraction. The results also suggest that for higher capsule loadings, longer healing times (>14 days) should result in higher healing efficiency and that experiments conducted at longer healing times could be used to further decouple the influence of solvent plasticization from other physical and chemical effects.

## Figures and Tables

**Table 1:** List of chemicals and structures used in this work.

Chemical Name	Chemical Structure	Abbreviation
Diglycidyl ether of bisphenol A	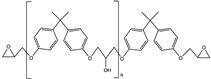	EPON 828
Furfuryl glycidyl ether	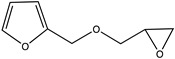	FGE
Biscyclohexanamine	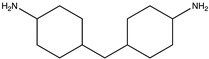	PACM
1,6′-bismaleimide-(2,2,4-trimethyl)hexane	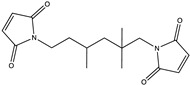	MMI-2
Phenyl acetate	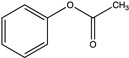	PA
